# Inhibitory effect of eugenol on seed germination and pre-harvest sprouting of hybrid rice (*Oryza sativ*a L.)

**DOI:** 10.1038/s41598-017-04104-x

**Published:** 2017-07-13

**Authors:** Qijuan Hu, Cheng Lin, Yajing Guan, Mohamed Salah Sheteiwy, Weimin Hu, Jin Hu

**Affiliations:** 1 Seed Science Center, College of Agriculture and Biotechnology, Zhejiang University, Hangzhou, Zhejiang 310058 P. R. China; 2 Department of Agronomy, Faculty of Agriculture, Mansoura University, Mansoura, 35516 Egypt

## Abstract

Pre-harvest sprouting (PHS) is a constrain problem in hybrid rice production. The present study was conducted to investigate the inhibitory effect of eugenol on seed germination and PHS of hybrid rice variety (Qian You 1). The results showed that seed germination speed and the activities of α-amylase were inhibited by eugenol pre-soaking and these effects enhanced with the increasing of eugenol concentrations; while seedling growth was not negatively affected. In field trials, eugenol application caused a significant decline in PHS as compared with control, whereas no sustained inhibition in post-harvested seed germination was observed. The HPLC analysis indicated that eugenol raised the internal ABA content by 1–4 times more than control, and seeds treated with eugenol had relatively lower *OsABA8OH2* and higher transcript levels of *OsNCED2* expression during early stages of seed imbibitions. In addition, seed germinated faster after GA_3_ application than eugenol alone, and seed endogenous ABA content decreased obviously. It suggested that eugenol strongly delayed seed germination and the PHS in the field, which might be mainly due to the increased ABA contents caused by eugenol. However, the phenomenon of delayed germination and high ABA content caused by eugenol could be effectively recovered by exogenous GA_3_.

## Introduction

Rice is one of the most important staple foods worldwide. Hybrid rice varieties possess many remarkable advantages over conventional rice such as higher yield, stronger stress tolerance and better grain quality^1^. Nevertheless, there still exist some disadvantages in hybrid rice. For instance, in hybrid rice, seeds germinated easily in ears before timely field harvest under high humidity and appropriate temperature condition. Pre-harvest sprouting happened much easier during production of hybrid rice varieties than conventional ones^2–4^. Pre-harvest sprouting generally reduces the cereal yield and degrades seed quality for planting, resulting in severe economic losses in seed production^5^. Therefore, determining the ways and solutions to reduce or eliminate the pre-harvest sprouting is crucial for hybrid rice seed production.

Seed germination is a complex process involving physically metabolism and recovery, essential cellular events to enable the embryo to emerge, and preparation for subsequent seedling growth^6^. Whether seeds could germinate well is an important issue for agricultural production. Although seeds are expected to germinate rapidly for field establishment, pre-harvest sprouting is not what farmers and seed companies expected. In addition to genetic improvement, application of exogenous chemical compounds is considered as effective method to prevent pre-harvest sprouting. So far, some substances including plant hormones and plant growth regulators had been reported for pre-harvest sprouting inhibition, like abscisic acid (ABA), ethephon, maleic hydrazide, paclobutrazol and uniconazole, etc^7, 8^. Specifically, abscisic acid (ABA) exhibits strong inhibitory effect on seed germination, but was not widely applied in practice due to its high cost. For this reason, we tried to seek an economical and environmental friendly product for practically reducing pre-harvest sprouting of rice in replacement for traditional chemical compounds.

A type of natural plant extracts, eugenol, is the major constituent of clove (*Syzygium aromaticum* L.) essential oil and possesses various biological activities. Eugenol is extracted and widely applied in multiple products and has found application in various industries such as food flavoring, fragrance, pharmaceutical^9–11^. Recently, eugenol was reported to have special herbicidal activity for some specific grassy weeds and might be developed as a new herbicide product to be widely used practically^12^. Although the inhibitory effect of eugenol on seed germination has been reported in wheat^13^, up to now, the internal mechanism underlying the effects of eugenol for reducing the germination in hybrid rice is not completely clarified.

Therefore, a comprehensive analysis was performed to better understand the underlying mechanism of inhibitory effects of eugenol on germination of rice seed. Different concentrations of eugenol were applied to rice seeds to determine the inhibitory effect of eugenol on seed germination. Furthermore, related physiological index and subsequent seedling growth were analyzed. Finally, a time course assay of endogenous hormones amounts as well as corresponding metabolism gene expression profiles of ABA during seed imbibition processes was also studied at the molecular level. In addition, we also performed field trial to evaluate the influence of eugenol on reducing pre-harvest sprouting and seed quality of hybrid rice after harvest. Then an environmentally safe inhibitor and its appropriate concentration would be developed to effectively delay seed germination and to reduce the damage caused by pre-harvest sprouting of hybrid rice.

## Results

### Inhibitory effect of eugenol on germination and seedling growth of Qian You 1

Eugenol application decreased seed germination speed of hybrid rice, and the inhibitory effect enhanced with the increasing of eugenol concentrations (Table 1). The mean germination time (MGT) of hybrid rice seeds increased and a reverse trend in case of germination index (GI) was observed with the advancement of eugenol concentration. The application of eugenol with 2.0 g · L^−1^ concentration recorded the highest level of MGT and lowest value of GI. On the contrary, the lowest values of MGT and highest values of GI were observed in control. However, no significant differences in GP were observed among control and eugenol treatments, indicating that the inhibitory effect of eugenol just on seed germination speed other than final germination percentage.

**Table 1:** Effects of different concentrations of eugenol on seed germination and seedling characteristics of hybrid rice Qian You 1.

Treatment	MGT (d)	GP (%)	GI	Shoot Height (cm)	Root Length (cm)	Seedling Dry Weight (g)
Control (water)	2.93 ± 0.05a	96.0 ± 0.6a	17.0 ± 0.6a	6.96 ± 0.03ab	6.20 ± 0.05a	89.0 ± 2.6b
Eugenol (1.0 g · L^−1^)	3.04 ± 0.04a	97.0 ± 1.0a	16.8 ± 0.2a	7.28 ± 0.01a	6.41 ± 0.03a	94.7 ± 2.3ab
Eugenol (1.5 g · L^−1^)	3.05 ± 0.04ab	94.3 ± 2.2a	15.7 ± 0.4b	7.11 ± 0.02a	6.18 ± 0.08a	101.0 ± 3.6a
Eugenol (2.0 g · L^−1^)	3.19 ± 0.04b	95.7 ± 1.4a	15.4 ± 0.2b	6.32 ± 0.06b	6.86 ± 0.06a	90.7 ± 4.1ab

Each value represented the mean (n = 3); small letters following the values within each column indicated significant differences (α = 0.05, LSD) among control and eugenol treatments.

In addition, eugenol applications had no negative inhibitory effect on seedling growth as compared with control on day 14 (Table 1). Both highest shoot height and root length were recorded in 1.0 g · L^−1^ of eugenol treatment. Seedling dry weight was promoted by eugenol compared with control, the highest dry weight of whole seedling (101.0 mg) was obtained from the 1.5 g · L^−1^ of eugenol, while the lowest value (89.0 mg) was observed in control.

### Inhibitory effect of eugenol on α-amylase activity

**Figure 1:**
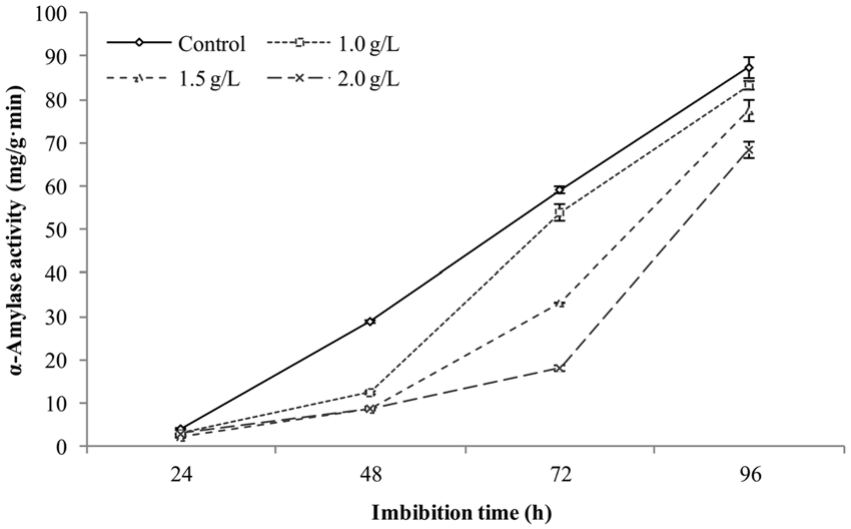
Effects of different concentrations of eugenol on α-amylase activities of Qian You 1. Control: seeds soaked in water; 1.0, 1.5 and 2.0 g · L^−1^: seeds soaked in the indicated concentrations of eugenol. The data were the means of three replications, and the standard deviation was indicated by bars.

As shown in Fig. 1, the α-amylase activity continuously increased from 24 to 96 h of seed imbibitions, eugenol applications had lower α-amylase activity as compared with control. In which, the high eugenol concentration (2.0 g · L^−1^) dramatically reduced the α-amylase activity at each sampling time during seed imbibition as compared with control and the other two eugenol treatments. After 96 h of imbibition, 1.0 and 1.5 g · L^−1^ of eugenol treatments had no significant differences in α-amylase activity as compared with control, however, 2.0 g · L^−1^ of eugenol still showed a significantly lower α-amylase activity than control.

### Effect of eugenol on PHS in the field and quality of freshly harvested seeds

**Figure 2:**
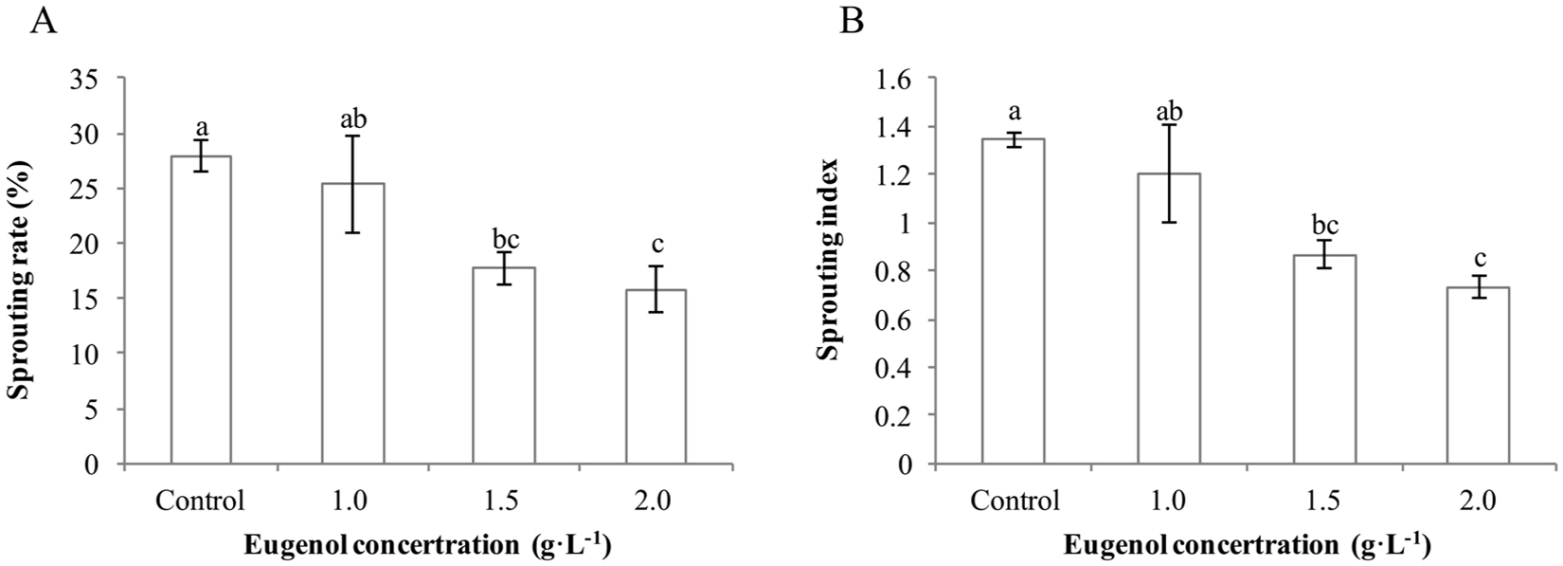
Effects of different concentrations of eugenol on the sprouting rate and sprouting index of Qian You 1 in the field. The data were the means of three replications, and the standard deviation was indicated by bars. Small letter (s) on top of bars indicated significant differences (α = 0.05, LSD) among control and different eugenol treatments at a certain imbibition time.

The effect of eugenol on the pre-harvest sprouting in the field was tested in 2013 year. As shown in Fig. 2, sprouting rate and sprouting index of seeds from the ears decreased obviously with the increasing of eugenol concentration as compared with control, and 1.5 and 2.0 g · L^−1^ of eugenol solutions reached significant levels. The sprouting rates of 1.0, 1.5 and 2.0 g · L^−1^ of eugenol treatments reduced by 9.3%, 36.5% and 43.4%, respectively; while the sprouting index decreased by 10.4%, 35.1% and 45.5%, respectively in comparison with control (Fig. 2). Whereas, no significant difference was observed among eugenol treatments and control in seed germination and seedling growth of freshly harvested seeds in laboratory (Table 2).

**Table 2:** Effects of different concentrations of eugenol on the germination and seedling growth of the freshly harvested hybrid rice seeds of Qian You 1 from the field trial.

Treatment	MGT (d)	GP (%)	GI	Shoot Height (cm)	Root Length (cm)	Seedling Dry Weight (g)
Control (water)	3.02 ± 0.03a	99.3 ± 0.7a	17.0 ± 0.2a	7.27 ± 0.87a	5.36 ± 0.43a	100.3 ± 5.5ab
Eugenol (1.0 g · L^−1^)	3.16 ± 0.13a	97.3 ± 1.8a	16.7 ± 0.9a	7.55 ± 0.11a	5.27 ± 0.29a	100.0 ± 3.1ab
Eugenol (1.5 g · L^−1^)	3.13 ± 0.04a	98.7 ± 0.7a	16.3 ± 0.2a	8.07 ± 0.16a	5.64 ± 0.38a	104.0 ± 1.0a
Eugenol (2.0 g · L^−1^)	3.18 ± 0.02a	97.3 ± 1.3a	16.0 ± 0.1a	7.16 ± 0.25a	5.27 ± 0.52a	89.3 ± 3.4b

Each value represents the mean (n = 3); small letters following the values within each column indicated significant differences (α = 0.05, LSD) among control and eugenol treatments.

### Effect of eugenol alone and eugenol-GA_3_ combined treatment on seed sprouting

**Figure 3:**
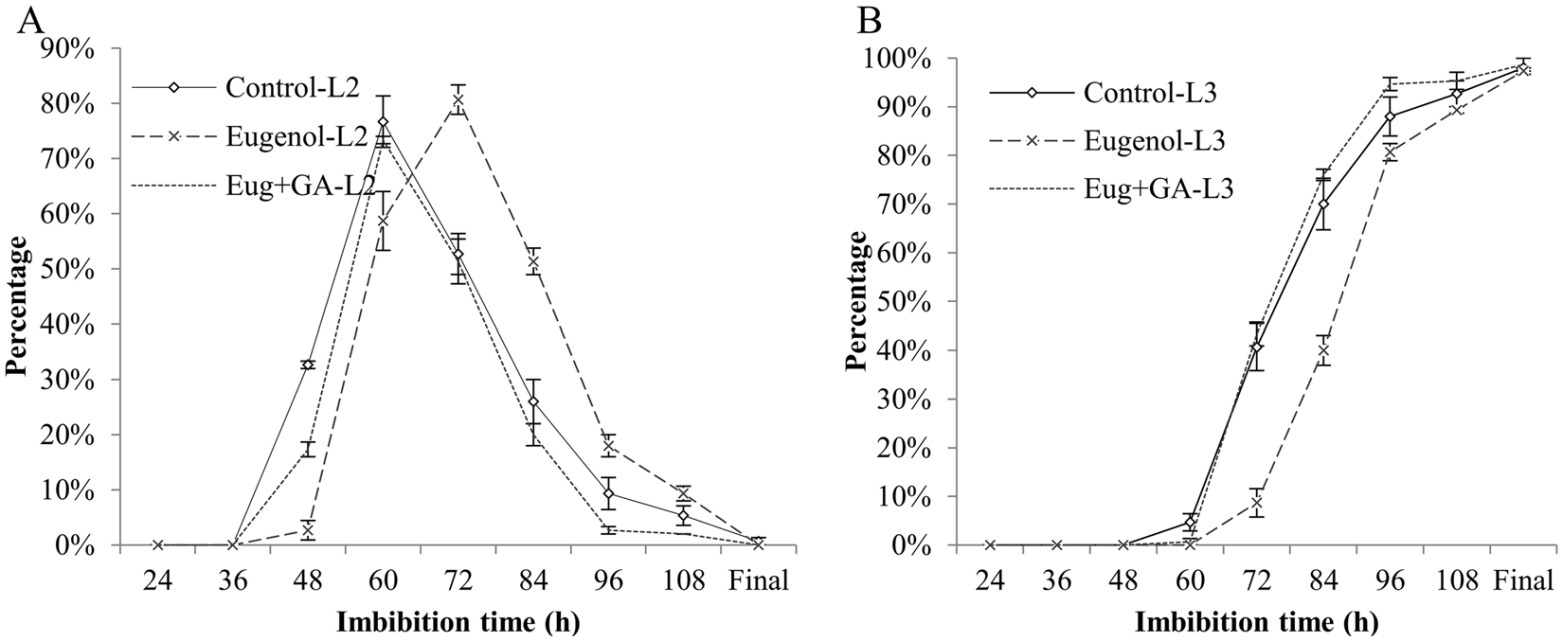
Effects of eugenol and eugenol-GA_3_ combined treatment on the percentage of Level 2 (**A**) and Level 3 (**B**) seeds of Qian You 1. Control: seeds soaked in water; Eugenol: seeds soaked in 2.0 g · L^−1^ of eugenol; Eug + GA: seeds soaked in 2.0 g · L^−1^ of eugenol + 50 mg · L^−1^ of GA_3_ mixed solution. L2: the radicle had extended to less than half the length of the whole seed; L3: the radicle length had extended to more than half the length of the whole seed. The data were the means of three replications, and the standard deviation was indicated by bars.

**Figure 4:**
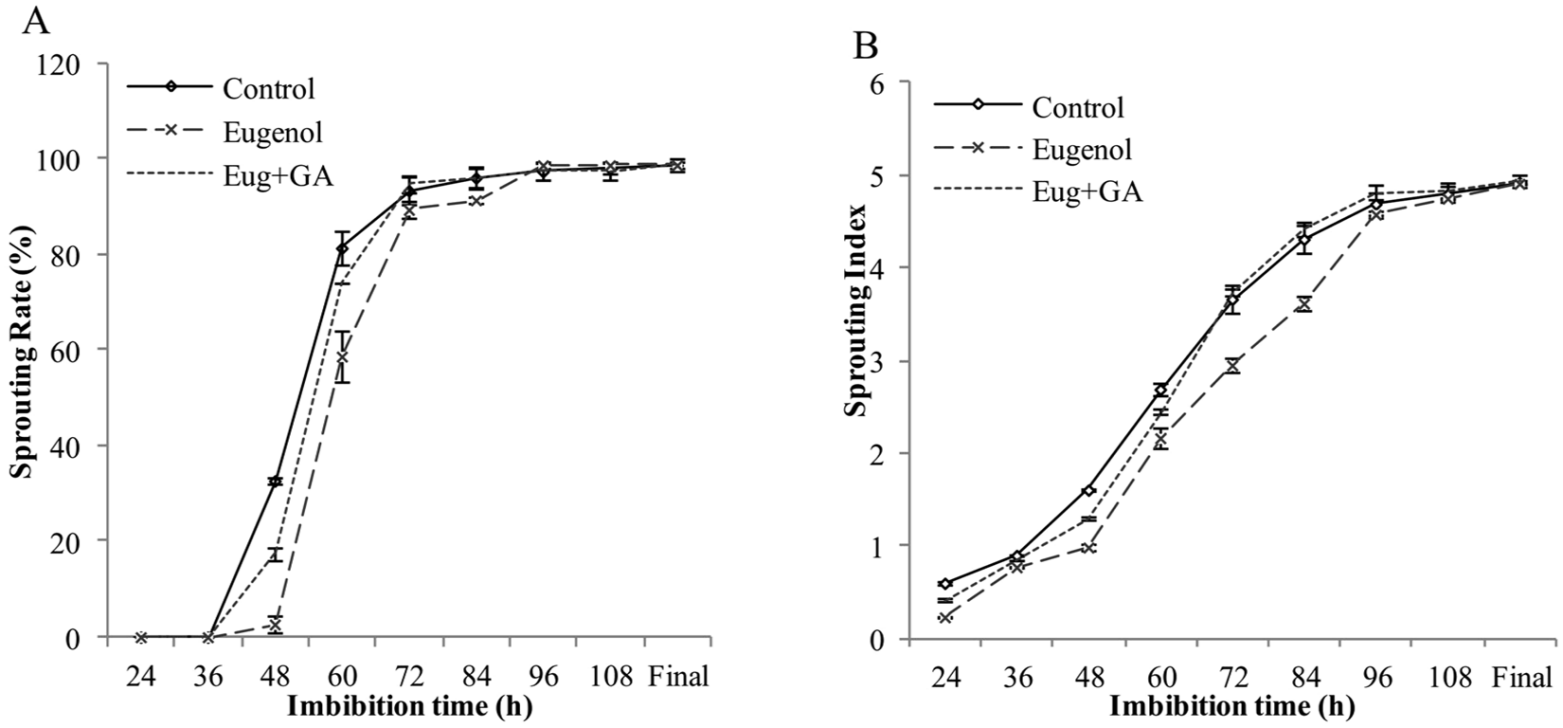
Effects of eugenol alone and eugenol + GA_3_ combined treatment on the sprouting rate (**A**) and sprouting index (**B**) of Qian You 1. Control: seeds soaked in water; Eugenol: seeds soaked in 2.0 g · L^−1^ of eugenol; Eug + GA: seeds soaked in 2.0 g · L^−1^ eugenol + 50 mg · L^−1^ of GA_3_ mixed solution. The data were the means of three replications, and the standard deviation was indicated by bars.

The results showed that 2.0 g · L^−1^ of eugenol delayed seed germination by 12 hours in comparison with control; while the eugenol-GA_3_ combined treatment had similar germination process with control did. It suggested that 50 mg · L^−1^ of GA_3_ could relieve the inhibitory effect of 2.0 g · L^−1^ of eugenol and recover seed germination rate to its original speed (Fig. 3). The maximum percentage of seeds with level 2 happened at 72 h in eugenol treatment; while those of control and combined treatment recorded at 60 h. Similar delayed effect was observed in seeds with level 3, which percentage at 72 h was already 40% in control and combined treatment, while only 7% in eugenol treatment. Moreover, as shown in Fig. 4, the eugenol treatment significantly delayed seed sprouting; however, both of the sprouting rate and sprouting index increased obviously after combined treatment and reached to the control levels at 72 h of imbibition. Similar results were observed in the germination test, which showed that the increased MGT and decreased GI caused by 2.0 g · L^−1^ of eugenol were effectively recovered by 50 mg · L^−1^ of GA_3_ (Table 3).

**Table 3:** Effects of eugenol alone and eugenol-GA_3_ combined treatment on seed germination of hybrid rice Qian You 1.

Treatment	MGT (d)	GP (%)	GI
Control (water)	3.01 ± 0.08b	98.7 ± 0.7a	17.44 ± 0.49a
Eugenol (2.0 g · L^−1^)	3.36 ± 0.02a	97.3 ± 0.7a	14.84 ± 0.11c
2.0 g · L^−1^ Eug + 50 mg · L^−1^ GA_3_	3.13 ± 0.03b	98.7 ± 1.3a	16.07 ± 0.05b

Each value represents the mean (n = 3); small letters following the values within each column indicated significant differences (α = 0.05, LSD) among control and eugenol treatments.

### Effects of eugenol alone and eugenol + GA_3_ combined treatment on endogenous ABA content

**Figure 5:**
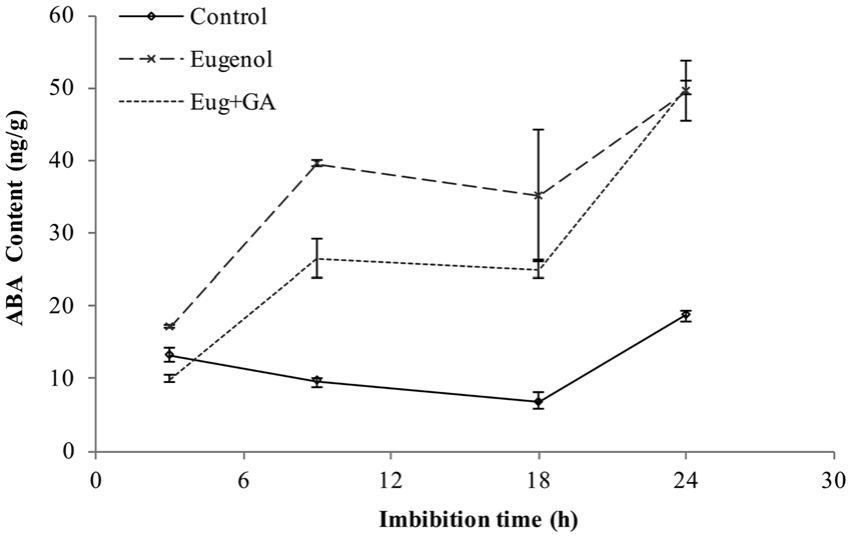
Changes of ABA contents in the presence of eugenol and eugenol + GA_3_ combined treatments during seed germination of Qian You 1. Control: seeds soaked in water; Eugenol: seeds soaked in 2.0 g · L^−1^ of eugenol; Eug + GA: seeds soaked in 2.0 g · L^−1^ eugenol + 50 mg · L^−1^ of GA_3_ mixed solution. The data were the means of three replications, and the standard deviation was indicated by bars.

In our study, internal ABA contents decreased from 3 h to 18 h of seed imbibition and always kept in lowest levels in control. The internal ABA contents were significantly improved by eugenol alone as compared with the control, which, however, declined significantly after seeds treated with eugenol + GA_3_ from 3 h to 18 h of seed imbibitions (Fig. 5). So it became reasonable that the delayed germination was associated with the enhancement of ABA synthesis at early seed imbibition.

### Effects of eugenol alone and eugenol + GA_3_ combined treatment on expression of ABA genes

**Figure 6:**
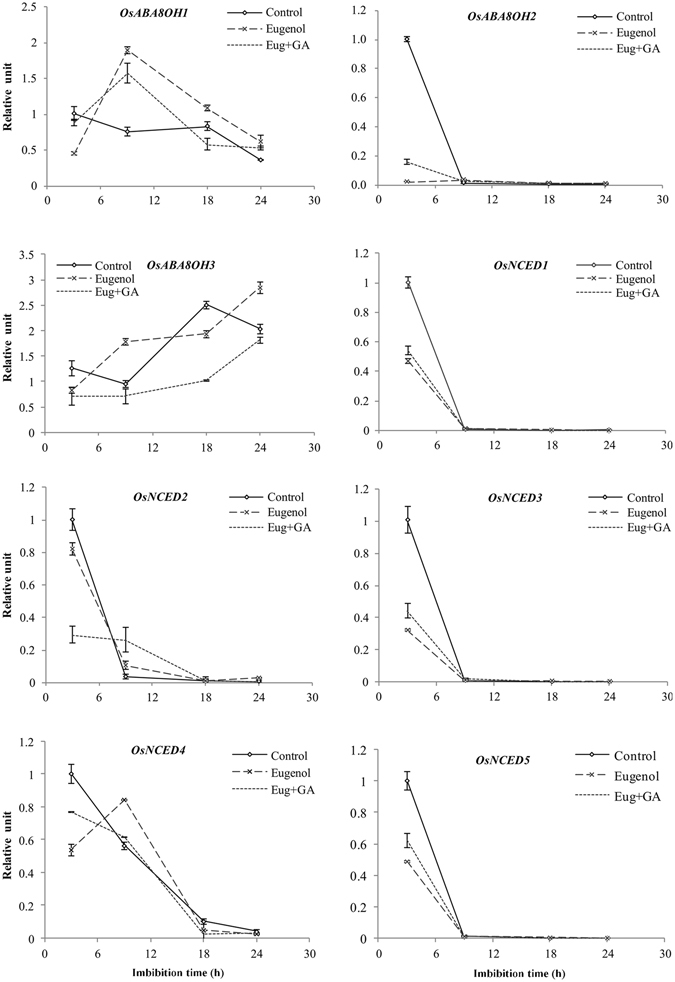
Changes of gene expression profiles of ABA metabolism during seed germination in Qian You 1. Gene expression profiles of ABA metabolism genes (*OsNCED1*, *OsNCED2*, *OsNCED3*, *OsNCED4*, *OsNCED5*, *OsABA8OH1*, *OsABA8OH2*, *OsABA8OH3*) were measured from 3 h to 24 h in rice cultivar Qian You 1. Control: seeds soaked in water; Eugenol: seeds soaked in 2.0 g · L^−1^ of eugenol; Eug + GA: seeds soaked in 2.0 g · L^−1^ of eugenol + 50 mg · L^−1^ of GA_3_ mixed solution. The data were the means of three replications, and the standard deviation was indicated by bars.

Expression levels of three members of *OsABA8OH* involved in ABA catabolism and five members of *OsNCED* involved in ABA synthesis were measured and each of them showed different expression patterns during seed imbibition (Fig. 6). The expression analysis of five *OsNCED*s showed that the *OsNCED1*, *OsNCED3* and *OsNCED5* had similarly changed trends in different treatments during seed imbibition. Their expression levels at 3 h were control > eugenol + GA_3_ > eugenol, which decreased dramatically from 3 h to 9 h and then kept very low levels from 9 h to 24 h. Differ from *OsNCED1*, *OsNCED3* and *OsNCED5*, the higher expressions of *OsNCED2* in eugenol and eugenol + GA_3_ treatments than in control were found during seed imbibitions except for 3 h. For *OsNCED4*, eugenol improved its expression from 3 h to 9 h and reached the highest level at 9 h as compared with control and combined treatment. The eugenol + GA_3_ decreased *OsNCED4* expression and had the lowest values except for 9 h from 9 h to 24 h of seed imbibition. For control, three *OsABA8OHs* expressions at 3 h were higher than those in eugenol and eugenol + GA_3_. *OsABA8OHs* transcript levels declined from 3 h to 9 h, in which the decrease of *OsABA8OH2* expression reached a very significant level. For eugenol treatment, *OsABA8OH1* and *OsABA8OH3* expressions increased obviously from 3 h to 9 h as compared with control, while the expression of *OsABA8OH2* always stayed in an extremely low level. For eugenol + GA_3_ combined treatment, *OsABA8OH1* level was higher than control but lower than eugenol. In contrast, *OsABA8OH2* level was significantly lower than control but a little higher than eugenol. The *OsABA8OH3* expressions in seeds treated by eugenol + GA_3_ stayed in lowest levels among three treatments. In addition, the expression ratio of *OsNCED2/OsABA8OH2* at 3 h in seeds treated with eugenol was obviously higher than those in eugenol + GA_3_ and control (Fig. S1 in Supplementary Data).

## Discussion

Eugenol is the major constituent of clove [*Syzygium aromaticum* (L.)] essential oil, which is extracted and widely applied in multiple chemical industries. Recently, eugenol was reported to have special herbicidal activity for some specific grassy weeds and might be developed as a new herbicide product to be widely used practically^12^. Its inhibitory effect on seed germination was also recorded in wheat for the first time. The results were consistent with the previous research which reported that 0.4 g · L^−1^ of eugenol could inhibit the germination of wheat seeds^13^. Whereas in the present study, the inhibitory concentration was 1.5–2.0 g · L^−1^ (four or five times of 0.4 g · L^−1^), it might due to that wheat seeds germinated in eugenol solution throughout the whole experiment, while hybrid rice seeds were just pre-soaked in eugenol solution for one hour and then germinated in water. It indicated as well that the applied concentration of eugenol should be determined according to the type of crop and the treatment methods on seed or seedling. Among the three eugenol treatments, 2.0 g · L^−1^ of eugenol recorded maximum in MGT and minimum in GI. Eugenol treatment resulted in no significant difference in the germination percentage and seedling length. Furthermore, seedling weight was even promoted by eugenol compared with control, indicating that eugenol delayed seed germination but don’t affect seedling growth negatively.

During seed germination, activation of α-amylase plays a predominant role in hydrolyzing endosperm starch to soluble forms as the principal energy for radical elongation and seedling growth^14, 15^. Therefore, higher α-amylase activity generally contributes to faster germination progress. In present study, the maximum value of α-amylase activity was found in control and the minimum was in 2.0 g · L^−1^ of eugenol, indicating that the synthesis of α-amylase was postponed by eugenol. The decrease of α-amylase activity caused by eugenol treatment was highly coincident with the delayed germination speed.

We further applied eugenol in the field trial to examine whether eugenol could reduce the pre-harvest sprouting in the field condition. As results show, the rice ears sprayed with eugenol had significantly lower sprouting rates and indexes than those treated with water, and the inhibitory effects increased with the increasing concentration of eugenol. In addition, there was no significant difference between the eugenol treatments and control in the germination test of seeds freshly harvested from the hybrid line, which suggested that the inhibitory effect of eugenol on seed germination could be relieved over a period of time. This phenomenon might be related to the volatile characteristic of eugenol and the similar results were found in the test on the seeds directly treated by eugenol in laboratory.

In order to examine whether the inhibitory effect of eugenol could be restored by gibberellins, a co-treatment of 2.0 g · L^−1^ of eugenol and 50 mg · L^−1^ of GA_3_ was applied to rice seeds. The results showed that 2.0 g · L^−1^ of eugenol delayed seed germination by 12 hours in comparison with control; while the eugenol-GA_3_ combined treatment had similar germination process with control did, which suggested that the delayed sprouting induced by eugenol could be relieved by GA_3_ to a certain degree.

Plant hormones especially ABA and GA profoundly influence the transition from seed dormancy to germination. The internal level of ABA in plants depends on the balance of its biosynthesis and catabolism. The decrease in ABA content is considered beneficial to seed germination^3^. In our study, internal ABA contents decreased from 3 h to 18 h of seed imbibition and always kept in lowest levels in control. This variation tendency was mostly consistent with the results reported by Jacobsen *et al*.^16^ who found that ABA level decreased during early stage of seed imbibitions^16^. In contrast, both eugenol alone and combined treatment increased ABA levels from 3 h to 9 h, and eugenol alone treatment raised more than combined treatment. It was suggested that GA_3_ could partially inhibit the increase of ABA content caused by eugenol.

ABA 8’-hydroxylase (ABA8′-OH) and 9-cis-epoxycarotenoid dioxygenase (NCED) are thought to play predominant roles in ABA catabolism and synthesis respectively^17, 18^. Compared with control, ABA accumulation occurred at the early stage of seed germination in seeds treated with eugenol and combined treatment might be due to a high synthesis of ABA or low catabolism of ABA. Biosynthesis of ABA in seeds is mainly regulated by the rate-limiting enzyme NCED. Induction of NCED increased ABA content and promoted possibly seed dormancy^19^. Except for 3 h, *OsNCED2* had higher expression levels in eugenol and combined treatment compared with control, which was consistent with the increased trends of ABA in seeds, suggesting that *OsNCED2* might contribute more to seed germination postponement induced by eugenol than the other *OsNCEDs* in our study. After eugenol treatment, the expression profile of *OsABA8OH2* at 3 h was significantly lower than control, which may contribute to lower catabolism of ABA and result in the higher ABA content. Based on the changed trends of *OsABA8OHs* and ABA content among the three treatments, *OsABA8OH2* was thought to have more contribution to internal ABA catabolism than *OsABA8OH1* and *OsABA8OH3* genes did. In addition, the results of *OsNCED2/OsABA8OH2* expression ratios in seeds imbibed for 3 h showed that eugenol has the higher ratio than the combined treatment and the Control (Fig. S1 in Supplementary Data). It highlighted that the delay of germination caused by eugenol was mainly induced by the suppression of ABA catabolism more than enhancement of ABA synthesis, which was similar with the study reported by Zhu *et al*.^8^ that the delay of rice seed germination induced by glucose mainly caused by suppressing *OsABA8OH* expression level^8^. Taken together, it concluded that eugenol could delay seed germination and reduce pre-harvest sprouting. However, there were no negative effects on seedling growth and quality of seeds harvested from eugenol treatments. Eugenol might promote content of ABA by mainly regulating relative genes involved in ABA catabolism. In addition, the inhibitory effects imposed by eugenol could be partially recovered by GA_3_.

## Methods

### Materials and seed treatment

Seeds of the hybrid rice variety Qian You 1 were obtained from the Zhejiang Nongke Seed Industry Co., Ltd., Hangzhou, P.R. China. Eugenol and Tween-20 were obtained from the Sinopharm Chemical Reagent Co., Ltd., Shanghai, P.R. China. ABA was obtained from Sigma-Aldrich Co., Ltd., St. Louis, Missouri USA. Eugenol was resolved in emulsifying agent Tween-20 to make eugenol solutions with different concentrations. A combined treatment consisted of eugenol and GA_3_ was made of equal volume of 2.0 g · L^−1^ of eugenol and 50 mg · L^−1^ of GA_3_. Before seed soaking, seeds for each treatment were sterilized with 0.1% NaClO for 15 min and then washed with tap water. Subsequently, seeds were treated with eugenol solutions or the combined treatment for 1 hour and dried over night at room temperature. Seeds were soaked in distilled water were used as control.

### Seed germination and seedling growth assay

Seeds were soaked in distilled water (control), 1.0 g · L^−1^, 1.5 g · L^−1^ and 2.0 g · L^−1^ of eugenol solutions for 1 hour and dried over night at room temperature. Then seeds were germinated in germination boxes (12 cm × 12 cm × 6 cm) containing 3 layers of moistened filter paper, and placed in germination chambers under a diurnal cycle of 8 h of light at 30 °C and 16 h of darkness at 20 °C for 14 days^20^. Seeds were considered as germinated when the radicle reached half the seed length^21^, and germinated seeds were counted daily until the 14th day of germination. The germination percentage (GP) was calculated on day 14. The germination index (GI) and mean germination time (MGT) were calculated as GI = Σ(Gt/Dt) and MGT = Σ(Gt × Dt)/ΣGt, where Gt is the number of germinated seeds on Day t, and Dt is the time corresponding to Gt in days. Root length and shoot height were manually measured in ten randomly selected seedlings on day 14. The dry weight of seedlings was measured after drying at 80 °C for 24 h^22^.

### Determination of α-amylase activity

Seed samples pre-treated with distilled water (control), 1.0, 1.5 and 2.0 g · L^−1^ of eugenol solutions were taken respectively at 24, 48, 72 and 96 h of imbibition, and were then quickly frozen in liquid nitrogen and stored at −80 °C. Each sample was ground into fine powder, homogenized with distilled water and then centrifuged at 5000 × g for 10 min. The supernatants were used for chromogenic reaction. The activity of α-Amylase was measured according to the 3, 5-dinitrosalicylic acid colorimetric (DNS) method as described by Li (2003)^23^ and calculated as enzyme activity = M × T/[R × W × t], where “M” is the maltose content (mg), “T” is the total volume of the extract, “R” is the volume of the extract used for the reaction, “W” is the weight of the seed sample and “t” is the reaction time.

### Field trial

The field trial was carried out from Sep 4, 2013 to Sep 17, 2013 at the experimental farm of Zhejiang University, Hangzhou, P.R. China. The hybrid rice cultivar, Qian You 1 was used to evaluate the inhibitory effects of eugenol on PHS of hybrid rice. Four rows of the female parent and one row of the male parent were grown. Control and three eugenol treatments, with three replications were arranged randomly in a complete randomized block design (CRBD). A total of 450 kg/ha of eugenol was applied to rice ears at early yellow maturity stage, and control was sprayed with an equal amount of water. One day after eugenol treatment, rain simulators sprayed water for 10 min every 30 min from 6:00 am to 6:00 pm till harvest time to establish a moist environment with an 85% relative humidity (the rice canopy temperature and humidity data in field from September 5 to September 17, 2015 was listed in Table S1 in Supplementary Data). Fifty ears harvested from the female plants of Qian You 1 in each replicate were used for the PHS analysis. As described by Hu *et al*.^24^, seeds threshed from spikes were classified into four levels to calculate sprouting rate and sprouting index. Level 0: un-germinated seeds; Level 1: the micropyle had broken, but the embryo had not yet penetrated; Level 2: the radicle had extended to less than half the length of the whole seed; Level 3: the radicle length had extended to more than half the length of the whole seed. Sprouting rate = (number of level 2 + number of level 3)/total number of seeds, sprouting index = (1 × number of level 1 + 3 × number of level 2 + 5 × number of level 3)/total number of seeds. The un-germinated seeds harvested from control and eugenol treatment were used for germination tests to verify the persistently inhibitory effects of eugenol.

### Seed sprouting Assay

In this portion, seeds were pretreated with distilled water (control), 2.0 g · L^−1^ of eugenol and the combined solution of eugenol and GA_3_ for 1 h. Seeds in each treatment were germinated as described for seed germination assay and counted every 12 h until all seeds germinated. At each time point, seeds were classified into four levels to calculate sprouting rate and sprouting index.

### Extraction and measurement of ABA

After imbibed in control, 2.0 g · L^−1^ of eugenol and eugenol + GA_3_ combined solution for 3 h, 9 h, 18 h and 24 h, seed samples were collected and quickly frozen in liquid nitrogen and stored at −80 °C. Approximately, 500 mg of powdered rice seeds were used for ABA measurement with HPLC following the method described by Qin (2013)^25^ and Tombesi *et al*.^26^ with some modification^25, 26^, 20 μL of extraction for each sample was injected into a High Performance Liquid Chromatography system consisting of Binary HPLC Pump (Model 1525, Waters) and Dual λ absorbance detector (Model 2487, Waters). Sample was separated through a C18 reversed-phase chromatographic column (SunFire, 5 μm, 46 × 250 mm, Waters) with flow velocity of 0.8 mL/min. ABA content were calculated according to the standard curve.

### Extraction of total RNA and qRT-PCR

Seeds were treated as described in extraction and measurement of ABA hormone. Total RNA was extracted from frozen seeds with an RNeasy Plant Mini Kit (Waryong, Beijing, China), and then 1 μg of RNA was reverse-transcribed into cDNA using a Hiscript QRT SuperMix for Qpcr (+gDNA wiper) (Vazyme, Nanjing, China). Transcript levels of each gene were measured by real-time PCR (qRT-PCR) using a LightCycler (Roche, Switzerland) and SYBR Green Master Mix (Vazyme, Nanjing, China) according to the manufacturers’ instructions. The cycling conditions included annealing at 95 °C for 120 s, followed by 40 cycles at 95 °C for 5 s and 60 °C for 1 min, and a final cycle of 95 °C for 10 s, 65 °C for 60 s and 97 °C for 1 s. qRT-PCR primers used in this study are listed in Table S2 in Supplementary Data. Transcript abundance was analyzed using the 2^−ΔΔCT^ method as described by Livak and Schmittgen (2000)^27^ and the experiments were repeated three times. The relative mRNA expression level was normalized to the amplification of a rice ACTIN gene (internal standard gene)^28^.

### Statistical analysis

Analysis of variance (ANOVA) of the obtained data was performed with SAS version 8.0 software (SAS Institute, Cary, NC). Prior to analysis, the percentage data were transformed according to *y* = arcsin [sqr (×/100)]. When a significant difference occurred in the treatments, the least significant difference was calculated (α = 0.05, LSD).

## Electronic supplementary material

Supplementary data
