# Supplementary material for: Inhibitory effect of eugenol on seed germination and pre-harvest sprouting of hybrid rice (Oryza sativa L.)
Source: Sci Rep. 2017 Jul 13;7:5295. doi: 10.1038/s41598-017-04104-x (PMC5509651; doi:10.1038/s41598-017-04104-x)
Supplement: Supplementary file 1 — Supplementary data [file 41598_2017_4104_MOESM1_ESM.doc]

**Inhibitory effect of eugenol on seed germination and pre-harvest sprouting of hybrid rice (*Oryza sativ*a L.)**

Hu Qijuan^1^, Lin Cheng^1^, Guan Yajing1,[[1]](#footnote-2)*, Sheteiwy Mohamed Salah^1,2^, Hu Weimin^1^, Hu Jin1,[[2]](#footnote-3)*

Table 1S. Rice canopy temperature and humidity data in field from September 5 to September 17, 2013

| Day | Sep 5th | Sep 6th | Sep 7th | Sep 8th | Sep 9th | Sep 10th | Sep 11th | Sep 12th | Sep 13th | Sep 14th | Sep 15th | Sep 16th | Sep 17th |
| --- | --- | --- | --- | --- | --- | --- | --- | --- | --- | --- | --- | --- | --- |
| Mean Temperature (°C) | 22.3 | 20.2 | 21.6 | 24.8 | 28.7 | 29.9 | 26.9 | 27.8 | 30.7 | 32.0 | 28.0 | 26.9 | 29.4 |
| Mean Humidity (%) | 80.1 | 93.8 | 93.8 | 89.7 | 86.7 | 86.1 | 92.0 | 91.6 | 81.9 | 73.7 | 85.2 | 80.8 | 75.0 |

Table 2S. Primers used in qRT-PCR analysis of genes involved in ABA metabolism in rice seeds

| Gene name | Sequences (5' to 3') |
| --- | --- |
| *Actin* | Forward 5' CGACCACCTTGATCTTCATGCTGCTA 3' |
|  | Reverse 5' CTTCATAGGAATGGAAGCTGCGGGTA 3' |
| *OsNCED1* | Forward 5' AGCCTCGGTCTTCCAATTTT 3' |
|  | Reverse 5' CACCCAACACAAAAGCTACG 3' |
| *OsNCED2* | Forward 5' GGTATGGAAACGAGGATAGTGGTT 3' |
|  | Reverse 5' TGCTTATTGTTGTGCGAGAAGTTC 3' |
| *OsNCED3* | Forward 5' AACCATCCAAACCGACGA 3' |
|  | Reverse 5' TGCCGAGAGAGAGAGAGTGTG 3' |
| *OsNCED4* | Forward 5' AACCATCCAAACCGACGA 3' |
|  | Reverse 5' TGCCGAGAGAGAGAGAGTGTG 3' |
| *OsNCED5* | Reverse 5' CGCCATCACCGAGAACTT 3' |
|  | Reverse 5' CTCGCCGAGCTTGAACAC 3' |
| *OsABA8OH1* | Reverse 5' TCAACACCTTCCAAGAGATGAA 3' |
|  | Reverse 5' ATCTCCTCCTCCCCGAAG 3' |
| *OsABA8OH2* | Reverse 5' CTACTGCTGATGGTGGCTGA 3' |
|  | Reverse 5' CCCATGGCCTTTGCTTTAT 3' |
| *OsABA8OH3* | Reverse 5' TGGGTTGGCCTTACATCG 3' |
|  | Reverse 5' TGAAGATCTCGCCGTACCTC 3' |


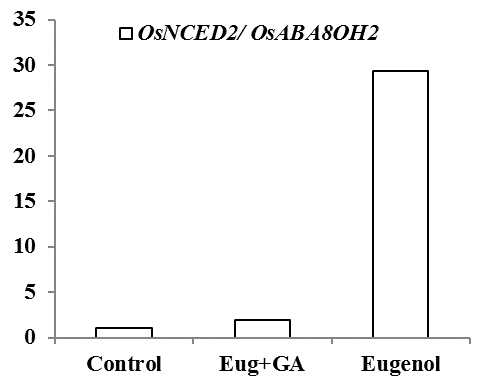


**Figure 1S.** The expression ratio of *OsNCED2/OsABA8OH2* in seeds imbibed for 3h after three treatments in hybrid rice Qian You 1.

Control: seeds soaked in water; Eugenol: seeds soaked in 2.0 g·L^-1^ of eugenol; Eug+GA: seeds soaked in 2.0 g·L^-1^ of eugenol+50 mg·L^-1^ of GA_3_ mixed solution. The data were the means of three replications, and the standard deviation was indicated by bars.

1. ^*^ Corresponding Author: E-Mail: [vcguan@zju.edu.cn](mailto:vcguan@zju.edu.cn); Phone: 0086-571-88982318 [↑](#footnote-ref-2)
2. ^*^ Corresponding Author: E-Mail: [jhu@zju.edu.cn](mailto:jhu@zju.edu.cn); Phone: 0086-571-88982318 [↑](#footnote-ref-3)
